# *De novo* assembly and characterization of the transcriptome in the desiccation-tolerant moss *Syntrichia caninervis*

**DOI:** 10.1186/1756-0500-7-490

**Published:** 2014-08-03

**Authors:** Bei Gao, Daoyuan Zhang, Xiaoshuang Li, Honglan Yang, Andrew J Wood

**Affiliations:** 1 Key Laboratory of Biogeography and Bioresource in Arid Land, Xinjiang Institute of Ecology and Geography, Chinese Academy of Sciences, Urumqi 830011, China; 2 University of Chinese Academy of Sciences, Beijing 100049, China; 3 Department of Plant Biology, Southern Illinois University-Carbondale, Carbondale 62901-6509, IL, USA

**Keywords:** Biological soil crust, Desiccation, *Physcomitrella*, Stress, *Syntrichia*, Transcriptome

## Abstract

**Background:**

*Syntrichia caninervis* is a desiccation-tolerant moss and the dominant bryophyte of the Biological Soil Crusts (BSCs) found in the Mojave and Gurbantunggut deserts. Next generation high throughput sequencing technologies offer an efficient and economic choice for characterizing non-model organism transcriptomes with little or no prior molecular information available.

**Results:**

In this study, we employed next generation, high-throughput, Illumina RNA-Seq to analyze the poly-(A) + mRNA from hydrated, dehydrating and desiccated *S. caninervis* gametophores. Approximately 58.0 million paired-end short reads were obtained and 92,240 unigenes were assembled with an average size of 493 bp, N50 value of 662 bp and a total size of 45.48 Mbp. Sequence similarity searches against five public databases (NR, Swiss-Prot, COSMOSS, KEGG and COG) found 54,125 unigenes (58.7%) with significant similarity to an existing sequence (E-value ≤ 1e-5) and could be annotated. Gene Ontology (GO) annotation assigned 24,183 unigenes to the three GO terms: Biological Process, Cellular Component or Molecular Function. GO comparison between *P. patens* and *S. caninervis* demonstrated similar sequence enrichment across all three GO categories. 29,370 deduced polypeptide sequences were assigned Pfam domain information and categorized into 4,212 Pfam domains/families. Using the PlantTFDB, 778 unigenes were predicted to be involved in the regulation of transcription and were classified into 49 transcription factor families. Annotated unigenes were mapped to the KEGG pathways and further annotated using MapMan. Comparative genomics revealed that 44% of protein families are shared in common by *S. caninervis*, *P. patens* and *Arabidopsis thaliana* and that 80% are shared by both moss species.

**Conclusions:**

This study is one of the first comprehensive transcriptome analyses of the moss *S. caninervis*. Our data extends our knowledge of bryophyte transcriptomes, provides an insight to plants adapted to the arid regions of central Asia, and continues the development of *S. caninervis* as a model for understanding the molecular aspects of desiccation-tolerance.

## Background

Biological soil crusts (BSCs) form at the surface of desert soils [1] and play an important role in arid and semi-arid ecosystems around the world [2,3]. BSCs are comprised of a number of organisms including cyanobacteria, green algae, fungi, liverworts, lichens and mosses [4]. Organisms found in BSCs have developed a suite of adaptive mechanisms that permit the avoidance of water loss and/or the survival of complete dehydration (i.e. desiccation) [3-7]. Desiccation-tolerant organisms have been observed among the three domains of life (Archaea, Bacteria and Eukarya) and the phenomenon has been extensively studied in cyanobacteria and plants [3,8,9]. Among land plants, desiccation-tolerance is rare in angiosperms and common in mosses [8-10]. More than 200 moss species have been experimentally verified to be desiccation-tolerant [9] and *Tortula ruralis* (=*Syntrichia ruralis*) is the model species for understanding the molecular aspects of vegetative desiccation-tolerance in mosses [6-11]. Desiccation-tolerant mosses are a key component of BSCs and *Syntrichia* ssp. are the dominant moss of both the Mojave [12] and Gurbantunggut deserts [13]. The Gurbantunggut desert (Xinjiang, China) is one of the major arid regions of central Asia with an area of 48.8 thousand km^2^, an average yearly precipitation of ~80 mm and mean annual pan evaporation of ~2607 mm [13-15]. Our research groups are interested in *Syntrichia caninervis*, a desiccation-tolerant moss and the dominant bryophyte of the Gurbantunggut desert BSC.

*S. caninervis* undergoes unpredictable cycles of dehydration and rehydration, and is frequently exposed to elevated temperature and higher amounts of UV-irradiation [16]. *S. caninervis*, like *T. ruralis*, can lose 90% of their protoplasmic water and subsequently rehydrate with no evidence of damage to the plasma membranes or chloroplasts [14,17]. A common feature of desiccation-tolerant mosses is the rapid recovery of photosynthesis and the rapid re-establishment of a positive carbon balance following rehydration [18,19]. Similar to other desiccation-tolerant mosses, rehydrated *S. caninervis* rapidly restores PSII activity in the context of enhanced Chl synthesis and the reorganization of PSII [16,19]. Following rehydration, *S. caninervis* has been shown to rapidly adjust leaf angle thereby maximizing net photosynthetic gain and minimizing water loss [20]. Successive cycles of rehydration/dehydration also have been shown to augment the surface wax content in *S. caninervis* leaves [21]. In addition to desiccation-tolerance, *S. caninervis* gametophores are extremely tolerant to heat and are able to regenerate following exposure to 120°C for 30 min [22].

The moss *Physcomitrella patens* is an important experimental model [23] and was the first published genome from a non-angiosperm, land plant [24]. The *P. patens* genome sequence allowed genome-wide analysis and demonstrated the utility of bryophyte genomes for the identification and characterization of plant genes [25-28]. However, *P. patens* is a mesic moss [9] and cannot survive desiccation [29]. As a key BSC species, *S. caninervis* is an attractive model for the study of desiccation tolerance and good candidate for -omic sequencing and analysis. Transcriptome sequencing is one of the most important tools for gene discovery and the identification of expression patterns [30-33]. Bryophyte transcriptomes has been generated and characterized from a number of species including *Marchantia polymorpha*[34], *Pohlia nutans*[35], *T. ruralis*[36,37] and *P. patens*[32,33]. Next generation high throughput sequencing technologies offer an efficient and economic choice for characterizing non-model organism transcriptomes with little or no prior molecular information available. Next generation sequencing platforms, such as Illumina, Roche 454 and SOLiD [38], have dramatically improved the efficiency of gene discovery and make it possible to detect low abundant transcripts [39,40].

In this study, we generated a global transcriptome assembly from *S. caninervis* using the Illumina HiSeq™ 2000 sequencing platform. Preliminary gene annotations of function, classification and metabolic pathways were obtained by searching public protein databases. We performed GO-based comparison with *P. patens*, comprehensive annotation of transcription factors and generated a MapMan metabolic pathway. Analysis of the *de novo* assembled transcriptome will provide a better understanding of the mechanisms associated with dehydration, the phenomena of vegetative desiccation-tolerance and identify a core set of abiotic stress-related transcripts.

## Results and discussion

### Illumina paired-end sequencing and *de novo* transcriptome assembly

**Figure 1:**
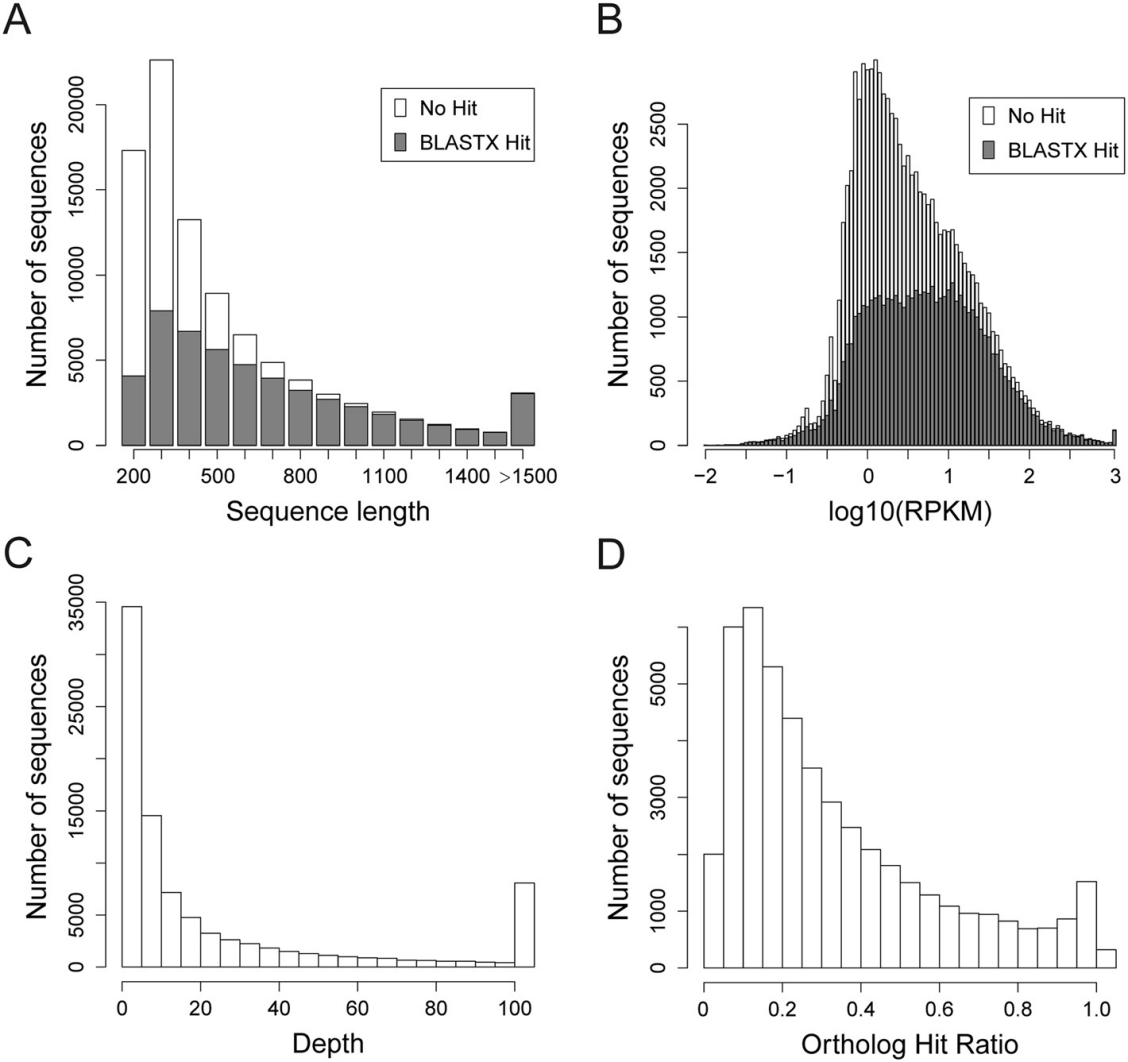
**Overview of the *S. caninervis *transcriptome sequencing and assembly. (A)** Histogram of the length of unigenes that returned BLASX hits in public protein databases and the sequences with no hit. **(B)** Distribution of unigene RPKM values for identified protein coding sequences and putative non-coding sequences. **(C)** Histogram of the average read depth for unigenes. Sequencing depth values above 100× was binned. **(D)** Ortholog hit ratio analysis for *S. caninervis* unigene sequences.

To generate a broad survey of transcripts associated with the *S. caninervis* dehydration/rehydration cycle, a cDNA library was constructed from mRNAs extracted from various dehydration and rehydration stages. Raw Illumina sequencing reads were quality and adapter trimmed to yield a total of 58,031,432 paired-end short reads comprising of 4.64 Gb of nucleotide data from a single sequencing run. The Q20 percentage was 97.55% and the GC content was 55.09% when assessing the cleaned reads with no ambiguous bases. *De novo* transcriptome assembly was performed using Trinity [41] generating 162,865 contigs with an average length of 288 bp and the N50 value was 429 bp. After final paired-end read mapping and clustering, 92,240 unigenes were assembled with an average size of 493 bp, N50 of 662 bp and a total size of 45.48 Mbp (Table 1). The transcript abundance of unigenes was evaluated relative to sequence length and RPKM value (Figure 1A, 1B). All the assembled unigenes longer than or equal to 150 bp were retained for further analysis. An average sequencing depth of 51× for the final unigene assembly was achieved (Figure 1C).

**Table 1:** Summary of sequence assembly after Illumina sequencing

Sequenced reads	Total number	58,031,432
	Total read length (bp)	4,642,514,580
	Reads length	90 + 70
	GC content	55.09%
	Q20 persentage	97.55%
Contigs	Total number	162,865
	Total length (bp)	46,952,370
	Mean length (bp)	288
	Contig N50 (bp)	429
Unigenes	Total number	92,240
	Total length (bp)	45,480,162
	Mean length (bp)	493
	Unigene N50 (bp)	662
	Minimum length (bp)	150
	Maximum length (bp)	4,909

Using BLASTX, we compared the number of base pairs in the unigene hit region to the total length of the open reading frame [42] from the best-matching protein sequence from *P. patens*[43]. The overall distribution of the ortholog hit ratio (OHR) for the assembled *S. caninervis* unigenes is depicted in Figure 1D. A total of 47,559 *S. caninervis* unigenes returned BLASTX hits with *P. patens* proteins with an OHR average of 33%. 4,116 unigenes (8.7%) had an OHR ≥ 0.8 and 10,762 unigenes (22.6%) had an OHR ≥ 0.5. Using this metric, more than one fifth of the putative *P. patens* orthologs captured within the *S. caninervis* transcriptome covered at least 50% of the predicted ORF. Similar analysis of Coelacanthiformes [44], Cypriniformes [45], Hemiptera [46] and Lepidoptera [42] have obtained ortholog hit ratios that range from 35-to- 72%. Similar to BLASTX hit ratios (Figure 1A), the ortholog hit ratio is also strongly governed by the length of unigenes. For unigenes longer than 1,500 bp average OHR = 0.68 while for unigenes shorter than 200 bp average OHR = 0.14. This data suggests that the sequencing data is suitable for further analysis.

### Annotation of the *S. caninervis* transcriptome

**Figure 2:**
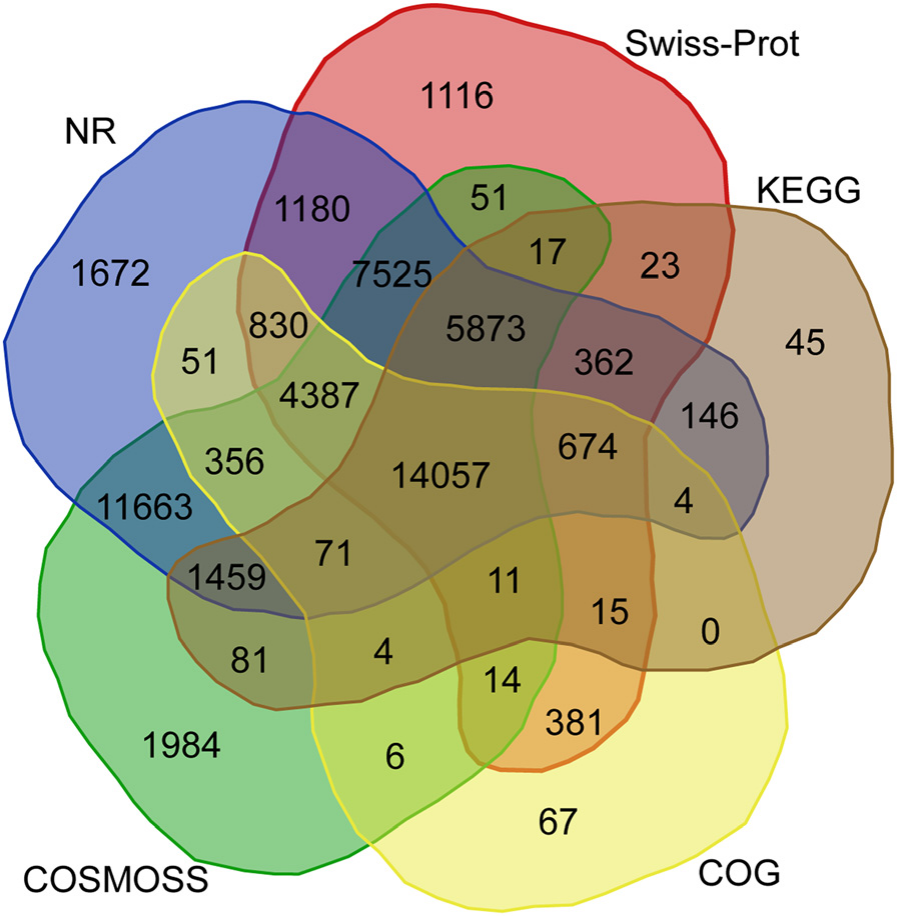
**Venn diagram showing the BLASTX results of the *S. caninervis *transcriptome against five protein databases.** Using BLASTX search, *de novo* reconstructed unigene sequences were queried against the following public databases: NCBI-NR, Swiss-Prot, COSMOSS, KEGG and COG. The number of transcripts that have significant hits (E-value ≤ 1e-5) against the five databases is shown in each intersection of the Venn diagram.

For annotation and classification of the assembled unigenes, we conducted sequence similarity searches against five public protein databases: NCBI nr protein database (NR), Swiss-Prot, COSMOSS v1.6 (*P. patens* proteins) [43], Kyoto Encyclopedia of Genes and Genomes (KEGG) and Clusters of Orthologous Groups (COG). Of all the 92,240 unigenes with length ≥ 150 bp, 54,125 (58.7%) revealed significant similarity (E-value ≤ 1e-5) with existing records in at least one of the five databases (Figure 2 and Additional file 1). Among the annotated unigenes, 51,938 (96%) unigenes returned a positive BLASTX hit with deduced polypeptides within either the NR or Swiss-Prot database. However, 38,115 unigenes (41.3%) have no significant match with any deposited sequence and are considered unknowns. Transcriptome annotation in other bryophyte species have obtained similar results with the percentage of unknown sequences greater than 40%: *T. ruralis* (40.3%) [36], *P. patens* (42%) [47], *Marchantia polymorpha* (43%) [34] and *Pohlia nutans* (82%) [35]. 14, 057 *S. caninervis* transcripts had significant similarity to deduced polypeptides in each of the five databases. 11,663 had significant similarity to sequences from both the NR and moss-specific COSMOSS databases. 4,884 unigenes had significant similarity to a single database: COSMOSS (1984 unigenes), NR (1672 unigenes), Swiss-Prot (1116 unigenes), KEGG (45 unigenes) and COG (67 unigenes).

**Figure 3:**
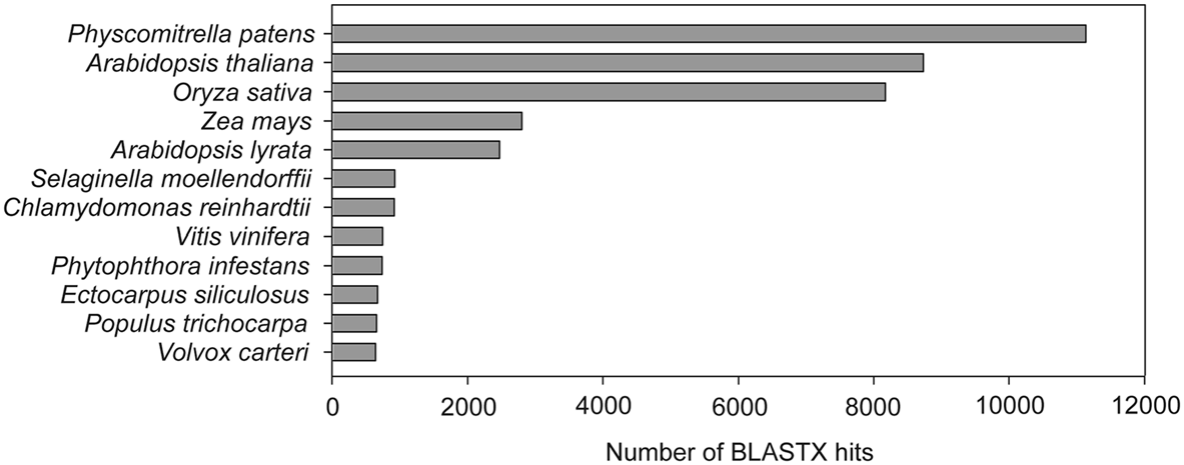
**Species distribution of the top BLASTX hits obtained using the *S. caninervis *transcriptome.** Top scoring BLASTX hits against the NCBI-NR protein database are depicted. The number of BLAST hits per species is shown on the x-axis. The 12 most represented species with proportions of more than 1% are shown in this graph.

Sorting the BLASTX hits by species revealed the top three species to be *P. patens* (11,136), *A. thaliana* (8733) and *O. sativa* (8173) (Figure 3). *P. patens* accounted for 22% of the identified unigenes and the three top-hit species accounted for more than 55% of the identified unigenes. The remaining 45% is distributed among members of the algae, ferns and angiosperms. It is important to note that several stress-related sequences have been identified and extensively studied in the closely related species *T. ruralis*[36]; however, due to the limited number of deduced polypeptide sequences present in the public databases, *T. ruralis* does not appear within the top hit species. These results indicate that a large number of moss-specific genes are present in the *S. caninervis* transcriptome (Figures 2 and 3). The presence of lineage-specific genes was a striking and dominant feature revealed in the reannotation of the *P. patens* genome (v1.6) [43]. 48% of all *P. patens* loci were clustered into *P. patens*-only clusters and about 22% (7,169) of all loci within *P. patens*-only clusters have no detectable homolog in any databases [43]. Among the annotated unigenes, 20,928 (40.1%) unigenes were assigned to the 25 COG categories (Table 2). Since some transcripts could be assigned to multiple COG functional categories, 39,756 total functional annotations were produced and all identified transcripts were grouped into one of the COG categories. “General function prediction”, “translation, ribosomal structure and biogenesis” were the two most represented categories (24% of all annotations), followed by “cell wall/membrane/envelope biogenesis” (9%), “transcription” (7%), “replication, recombination and repair” (7%) and “lipid transport and metabolism” (6%). Among the transcripts classified into specific COG functional categories, 2,618 unigenes (13%) were identified as “function unknown”. We hypothesize that these unknown and unclassified (Table 2) transcripts might represent species- and/or lineage-specific genes for adaptive innovations.

**Table 2:** **COG functional classification of *S. caninervis *** **transcripts**

**Description**	**Code**	**Unigenes (n)**
**Information storage and processing**		
RNA processing and modification	A	125
Chromatin structure and dynamics	B	342
Translation, ribosomal structure and biogenesis	J	4,202
Transcription	K	2,927
Replication, recombination and repair	L	2,876
**Cellular processing and signaling**		
Cell cycle control, cell division, chromosome partitioning	D	1,812
Cell wall/membrane/envelope biogenesis	M	1,774
Cell motility	N	348
Posttranslational modification, protein turnover, chaperones	O	2,825
Signal transduction mechanisms	T	2,069
Intracellular trafficking, secretion, and vesicular transport	U	1,249
Defense mechanisms	V	477
Extracellular structures	W	19
Nuclear structure	Y	9
Cytoskeleton	Z	496
**Metabolism**		
Energy production and conversion	C	1,782
Amino acid transport and metabolism	E	1,584
Nucleotide transport and metabolism	F	447
Carbohydrate transport and metabolism	G	2,573
Coenzyme transport and metabolism	H	685
Lipid transport and metabolism	I	1,247
Inorganic ion transport and metabolism	P	1,126
Secondary metabolites biosynthesis, transport and catabolism	Q	932
**Poorly characterized**		
General function prediction only	R	5,211
Function unknown	S	2,619

n = number of unigenes.

### Gene ontology annotation and comparison with the *P. patens* genome

**Figure 4:**
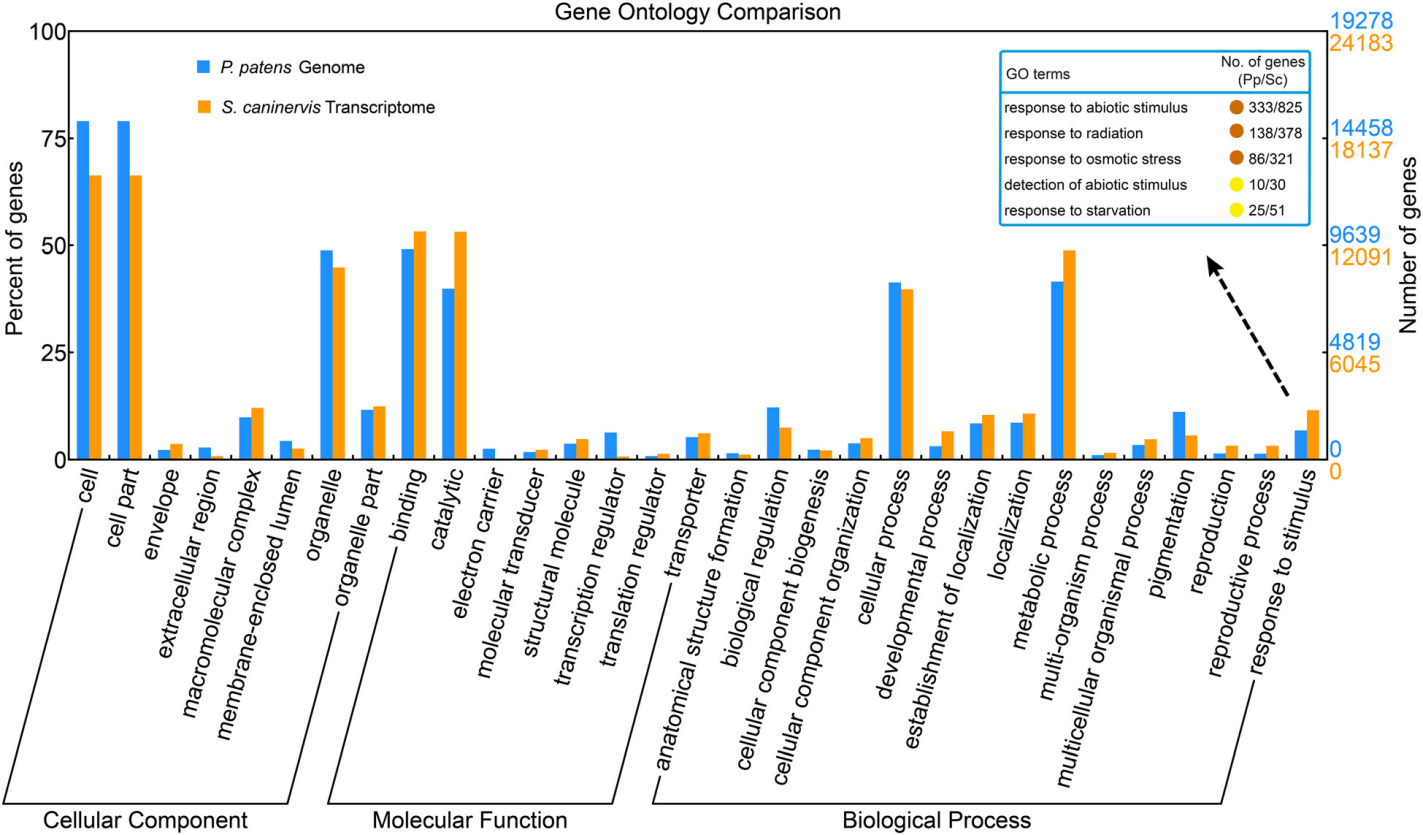
**Gene ontology classification and comparison between the mosses *P. patens *and *S. caninervis*.** Gene ontology annotation results of the genes from the *P. patens* genome and *S. caninervis* transcriptome were mapped to categories within the second level of GO terms. GO terms that contain more than 1% of total genes were included in this graph. Abiotic stress related subcategories of the term “response to stimulus” were shown in the box.

Gene ontology (GO) terms were assigned to each *S. caninervis* transcripts based on its best BLASTX hits in the NR database using the Blast2GO pipeline [48]. Of the 50,310 unigenes with NR annotation, 24,183 unigenes (48%) were assigned to one of three GO terms: Biological Process, Cellular Component or Molecular Function (Figure 4 and Additional file 2). Previous GO annotation of the *P. patens* genome assigned a functional annotation to 41% of the transcripts [49] and a subsequent reannotation increased the functional annotation to 58% [43]. GO comparison between *P. patens* and *S. caninervis* demonstrated similar sequence enrichment across all three GO categories. Transcripts were enriched for both *P. patens* and *S. caninervis* within the Cellular Component category (“cell”, “cell part” and “organelle”), the Molecular Function category (“binding” and “catalytic”) and the Biological Process category (“metabolic processes” and “cellular processes”, “response to stimulus”, “localization” and “establishment of localization”). Although enriched in both moss species, the “response to stimulus” category was significantly more enhanced in *S. caninervis* relative to *P. patens*. (2,775 vs. 1,335 unigenes) (Figure 4 and Additional file 2). Detailed analysis of the “response to stimulus” category revealed significant differences between the *S. caninervis* transcriptome compared with *P. patens* genome. Unigenes related to “response to radiation”, “osmotic stress”, “detection of abiotic stimulus” and “response to starvation” were significantly more represented in the *S. caninervis* transcriptome. Notably, the “translation regulator” category is significantly more represented in *S. caninervis*, while the “transcription regulator” category is less significantly represented.

Additionally, a hypergeometric statistical test was employed to identify over-represented (p-value < 0.05) GO categories present in the most abundantly expressed genes (RPKM ≥ 100) (Figure 1B). GO enrichment analysis on the most abundant unigenes demonstrated transcripts clustered in the “response to stress”, “abiotic stimulus”, “lipid metabolic process”, “generation of precursor metabolites and energy”, “membrane”, “thylakoid”, “plastid” and “ribosome” were significantly overrepresented as compared to the entire *S. caninervis* transcriptome (Additional file 3). Studies into the response to desiccation and rehydration of desiccation-tolerant mosses such as *S. caninervis* and *T. ruralis* have proposed that a constitutive protective mechanism and an active rehydration-induced recovery mechanism is employed [14,17]. Annotation of the *S. caninervis* transcriptome supports the hypothesis that transcripts related to transcriptional gene control, response to abiotic stress, photosynthesis, membrane integrity and translational regulation play an important role rehydration/dehydration cycle.

### Protein family assignment and transcription factor prediction

**Figure 5:**
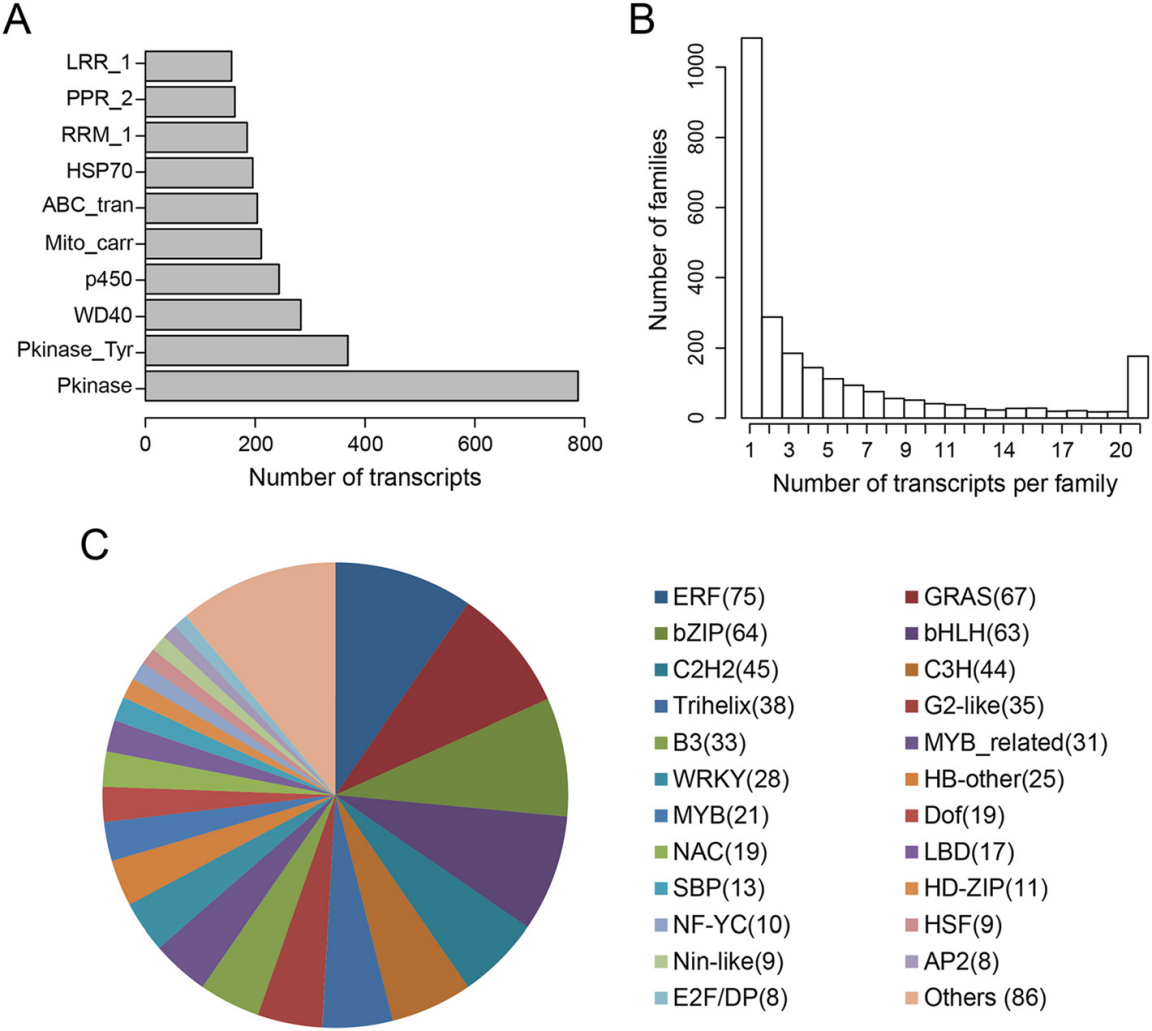
**Protein families and transcription factors in the *S. caninervis *transcriptome. (A)** The 10 most abundant protein families in the *S. caninervis* transcriptome. **(B)** Relationship between the occurrence of *S. caninervis* transcripts and the number of Pfam families in the *S. caninervis* transcriptome. **(C)** The 23 most abundant predicted transcription factor protein families. The number of members in each TF family is presented within the brackets. A total of 778 TFs were predicted and classified into 49 TF families (Additional file 1).

Identifying conserved domains present within a deduced polypeptide can provide insight into the function, regulation and/or localization of the predicted protein. An ORF was predicted for each *S. caninervis* unigene (see Methods) and the deduced polypeptide sequences were queried for the presence of protein motifs using the Pfam database [50]. 29,370 deduced polypeptide sequences were assigned Pfam domain information and categorized into 4,212 Pfam domains/families (Additional file 4). Pfam domains/families were ranked according to the frequency of occurrence of *S. caninervis* transcripts. The top 10 abundant domains/families are depicted in Figure 5A. The majority of the domains/families contained a small number of transcripts (i.e. 10 or less) and ranged from 1 to more than 700 transcripts per family (Figure 5B). “Protein kinase”, “protein kinase-tyrosine”, “WD40”, “leucine rich repeat”, “P450”, “ABC-containing” and “HSP70” are among the most abundant domain/families in *S. caninervis*. Protein kinases are known to function as an on/off switch and play a role in a multitude of cellular processes, such as metabolism and cell division [51]. WD40-repeat proteins are known to coordinate multi-protein complex assemblies and the proteins containing this domain are implicated in a variety of functions ranging from signal transduction and transcription regulation to cell cycle control, autophagy and apoptosis [52]. Proteins with leucine-rich repeat domains are frequently involved in the formation of protein–protein interactions [53]. P450 domain containing enzymes incorporate oxygen into small lipophilic compounds and play an important role in the biosynthesis of biopolymers and defense chemicals [54]. ABC-containing peptides are membrane-localized transport proteins that mediate the exchange of a broad range of molecules including metals, hormones lipids and secondary metabolites [55,56].

In order to more fully understand gene control and regulation in *S. caninervis*, all transcription factors were predicted according to the family assignment rules illustrated in PlantTFDB [57]. 778 unigenes were predicted to be involved in the regulation of transcription and were classified into 49 transcription factor families (Figure 5C and Additional file 1). The ERF (AP2-domain) transcription factor family is the most abundant TF families in *S. caninervis* and similar results were observed in *P. patens* as recorded in PlantTFDB [57]. AP2/ERF proteins have important functions in the transcriptional regulation of a variety of biological processes related to growth and development, abiotic stress tolerance and response(s) to a variety of environmental stimuli [58]. The remainder of the top 10 TFs are: GRAS, bZIP, bHLH, C2H2, C3H, Trihelix, G2-like, B3 and MYB-related. Transcription factor families with low gene number may play a unique role in gene control. *S. caninervis* contains a single VOZ (Vascular plant One Zinc finger protein) transcript and two CO (CONSTANS) transcripts. VOZ was characterized in *Arabidopsis* and homologues have been identified in the moss *P. patens*[59] but not in the liverwort *M. polymorpha*[34]. In *Arabidopsis*, VOZ genes have been implicated in the regulation of flowering time [60] and respond to both biotic and abiotic stress [61]. CO was also characterized in *Arabidopsis*[62] and plays an important role in photoperiod-regulated flowering. CO and CO-like genes are plant-specific and homologues have been identified in all flowering plants as well as in *P. patens*[63,64].

### Metabolic pathways

To survey genes involved in important metabolic pathways, annotated *S. caninervis* transcripts were mapped to the Kyoto Encyclopedia of Genes and Genomes (KEGG) pathways. A total of 22,842 unigenes (44%) were mapped to 119 KEGG metabolic pathway maps (Additional file 5). Among the 119 KEGG pathways, the pathways most represented by unigenes were “metabolic pathways” (5,730; 25%), “biosynthesis of secondary metabolites” (2,674; 12%), “ribosome” (1,891; 8%) and “plant-pathogen interaction” (1,310; 6%). These results are consistent with the hypothesis that moss transcriptomes reflect an enhanced versatility and mediate a variety of alternative metabolic pathways not observed in tracheophytes [25]. Examination of the colored KEGG maps demonstrated that we have captured almost all the genes required for the citrate cycle, photosynthesis, carbon fixation in photosynthetic organisms, flavonoid biosynthesis and the biosynthesis of unsaturated fatty acids. In addition, *S. caninervis* transcripts were annotated with MapMan bins and subsequently classified according to their major metabolic pathways and normalized expression level (Additional file 6). MapMan analysis showed that most biochemical pathways have been captured and demonstrated the diversity and completeness of the transcriptome. Consistent with the GO classification, the MapMan functional classification of metabolism showed high numbers and high expression levels of transcripts involved in lipids metabolism and the light reactions of photosynthesis.

### Orthologous relationship with model plants

**Figure 6:**
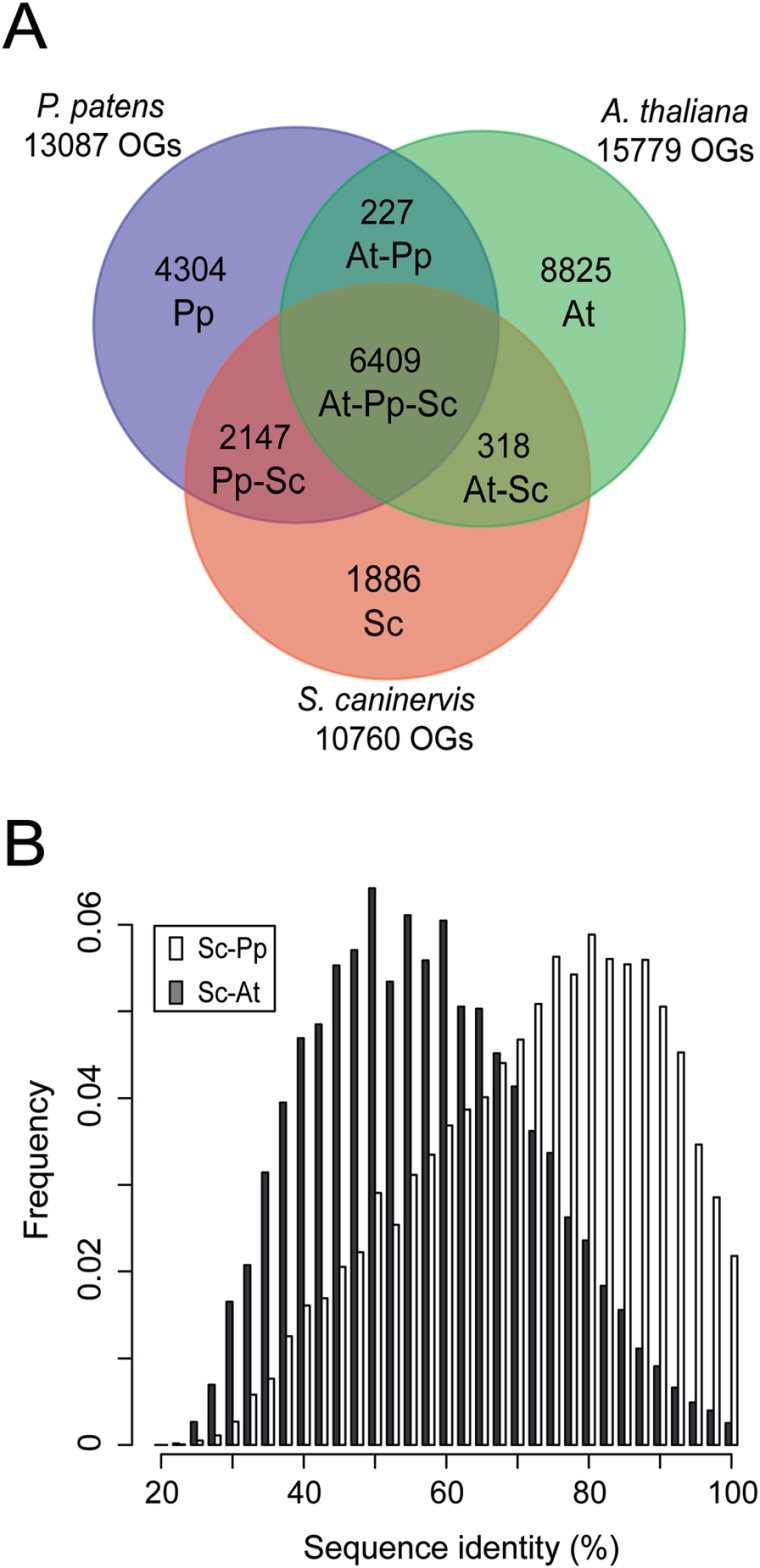
**Detection of homologous genes in mosses and *Arabidopsis*: comparison of orthologous gene groups and protein sequence identity. (A)** OrthoMCL was used to identify orthologous groups (OGs) among *S. caninervis* (Sc), *A. thaliana* (At) and *P. patens* (Pp). **(B)** Density plot of the protein identity between *S. caninervis* and the model plants.

Prior to analysis of Plant Ortholog Group (Plant OG) membership, proteins inferred from the *S. caninervis* transcriptome were clustered to construct a reference protein dataset comprised of 41,530 representative protein sequences. Clustering reduces the presence of redundant sequences and base miscall errors, but can also eliminate highly similar homeoalleles. To further investigate proteomic similarity with *P. patens* and *A. thaliana*, we clustered the three proteomes into protein families using OrthoMCL [65] (Figure 6A). Most (~80%) of the OrthoMCL-defined protein families in *S. caninervis* are in common with *P. patens*. Approximately 44% of the protein families from the tracheophyte, *Arabidopsis*, are in common with the two bryophytes. 65% of the protein families are present in *Arabidopsis*, 54% present in *P. patens* and 45% are present in *S. caninervis*, and 35% are found in common between *P. patens* and *S. caninervis. S. caninervis* has significantly more OGs in common with *P. patens* as compared to *Arabidopsis*. BLASTP comparison of the deduced polypeptides between *S. caninervis* and *P. patens* (Sc-Pp), and *S. caninervis* and *Arabidopsis* (Sc-At) is presented in Figure 6B. In both cases the sequence identity ranged from 20% to 100%. The Sc-Pp mean is 71% and the Sc-At mean is 56%. *S. caninervis* has not only significantly more OGs in common with *P. patens* but also greater sequence identity with *P. patens* as compared to *Arabidopsis*.

The data identifies several categories of transcripts: 1) sequences shared in common between mosses and an angiosperm, 2) sequences found in *Arabidopsis*, 3) sequences found in mosses, 4) sequences found in *P. patens* and 5) sequences found in *S. caninervis*. Our particular interest in desert plants led to a comparison of the *S. caninervis* and *T. ruralis* transcriptomes. *T. ruralis* is a model organism for studying stress-responsive gene control and a large number of cDNAs/ESTs/transcripts have been isolated and characterized [36,37]. We have mined the current *S. caninervis* transcriptomic database and identified homologues (>95% identical at the amino acid level) to many previously characterized *T. ruralis* transcripts including *Tr155*[66] and *Tr288*[67], *Elipa* and *Elipb*[68], *ALDH7B6*[69], *ALDH21A1*[70], *Vac1*[71], *TrDr1* and *TrDr2*[72] and the moss-retained *TrDr3*[73]. Identification of “desiccation related” homologues in both *T. ruralis* and *S. caninervis* support our long-standing hypothesis that desiccation-tolerant mosses proffer novel genes and gene products [37]. Further research is required to confirm the features and functions of these putative moss-specific and stress-tolerance related genes.

## Conclusions

In this study we present a global characterization of the *S. caninervis* transcriptome using next-generation, RNA-Seq technology. Using high-throughput sequencing technology, we have captured most of the transcripts expressed in moss gametophores during rehydration and desiccation. *De novo* transcriptome assembly generated 92,240 unigene sequences. GO annotation of the *S. caninervis* transcriptome and comparison with the *P. patens* genome demonstrates the enrichment of sequences related to transcriptional gene control, response to abiotic stress, and translational regulation. Our data extends our knowledge of bryophyte transcriptomes, provides an insight to plants adapted to the arid regions of central Asia, and continues the development of *S. caninervis* as a model for understanding the molecular aspects in desiccation-tolerance.

## Methods

### Sample collection, cDNA library construction and Illumina sequencing

*S. caninervis* gametophytes were collected from the Gurbantunggut Desert of Xinjiang Uygur Autonomous Region of China (44° 32′ 30″ N, 88° 6′ 42″ E) and harvested and stored as described previously [14]. Since 2003, this sand dune has been identified as a permanent research site. In this study, patches of *S. caninervis* inhabiting the biological soil crusts were collected in petri dishes and stored in an air-dried state for at least 1 week at room temperature. All samples used in the experiment were collected from the same site within a 10 m^2^ plot. Voucher specimens are maintained in the Department of Plant Biology, Southern Illinois University (Carbondale, IL). To obtain a comprehensive transcriptome assembly of *S. caninervis* transcripts during the dehydration-rehydration process, an equal mixture of total RNAs isolated from various dehydration and rehydration time points were used to construct the sequencing library. Dried gametophyte tissue samples were placed on filter paper in petri dishes and rehydrated using purified water for 24 hours. Gametophores were harvested after 24 h of rehydration. Gametophores were subsequently allowed to dry on an open bench (ca 25°C, RH =25%) [74] and samples (i.e. 100 mg FW) were harvested at 0.5, 1, 1.5, 2, 4, 6, 8, 10, 12 and 24 h.

Total RNAs isolated from all samples were quality and purity assessed and pooled together for RNA-Seq [75]. Total RNAs were extracted from *S. caninervis* gametophyte tissue samples using Trizol Reagent (Invitrogen, USA). The resulting samples were treated with DNase I to remove any genomic DNAs. RNAs were quantified using an Agilent 2100 Bioanalyzer and checked for RNA integrity using denaturing agarose gel electrophoresis. The cDNA library was created and sequenced according to the manufacturer’s instructions (Illumina) and sequencing was performed at Beijing Genome Institute (BGI) in Shenzhen, China. Briefly, beads with Oligo(dT) were used to isolate poly(A) + mRNA after total RNA was obtained. Fragmentation buffer was added for interrupting mRNA into short fragments. First-strand cDNA was synthesized using these short fragments as templates, along with reverse transcriptase and random hexamer primer. And the second-strand cDNA was synthesized using buffer, dNTPs, RNaseH and DNA polymerase I. The resulting double stranded cDNA was then subjected to end-repair using T4 DNA polymerase, DNA polymerase I Klenow fragment, and T4 polynucleotide kinase, and ligated to adapters using T4 DNA ligase. Short fragments were purified with QIAquick PCR purification kit and eluted with EB buffer. After agarose gel electrophoresis, the suitable fragments (200 ± 50 bp) were selected as templates for bridged PCR amplification. The Illumina cBOT was used for cluster generation following the manufacturer’s instructions, and the clustered flow cell was loaded onto the sequencing machine. cDNA library products were sequenced on an Illumina HiSeq™ 2000 system.

### Data filtering and *de novo* transcriptome assembly

Before assembly, clean reads of high quality were generated from the raw reads by removing adapter sequences, low-quality reads with ambiguous bases (‘N’), and reads with more than 10% of Q-values < 20 bases. All subsequent analyses were based on the clean reads. The quality of clean reads was further evaluated using NGS QC toolkit [76]. *De novo* transcriptome assembly was performed using Trinity [41] (release 2011-07-13). At the first step all clean reads were randomly clipped into 25-mers for assembly using de Bruijn graph algorithm, and we got the longest assembled fragments called contigs. Then paired-end reads were mapped back to contigs, using paired reads it is able to detect contigs from the same transcript as well as merge them. Finally, we obtained the sequences which cannot be extended on either end, and the resulting sequences were defined as unigenes. Only the unigene sequences longer than or equal to 150 bp were reserved for further analysis. Following assembly, unigenes were assigned an RPKM value [39] based on the number of uniquely mapped reads aligning to each unigene using SOAP [77] software (release 2.21).

### Evaluation of transcripts integrity

The “Ortholog Hit Ratio” method proposed by O’Neil et al. was used to determine how closely our sequences approached full-length transcripts [42]. The “ortholog hit ratio” metric computes the length of the putative coding region found in the newly assembled sequence divided by the full length of its top BLAST hit. Thus, an ortholog hit ratio of 1.0 may imply that a transcript has been assembled to its true full length. *P. patens* is the most well-studied bryophyte with a complete reference genome and comprehensively annotated gene sequences [43]. A blastx search against protein sequences inferred from *P. patens* genome (COSMOSS v1.6) was used to evaluate the quality of our assembled transcripts. For the purposes of this study, we consider each unigene and its best *P. patens* BLASTX hit with an E-value ≤ 1e-5 to be a putative ortholog. All top hits for *S. caninervis* transcripts were parsed and used to calculate ortholog hit ratios. If there are relative insertions in best hit *P. patens* proteins, this will tend to lower ortholog hit ratios, while ratios bigger than 1.0 likely indicate insertions in the query sequence relative to its top BLAST hit.

### Gene annotation and classification

For annotation and classification of the transcriptome, all assembled unigene sequences were subjected to BLASTX (E-value ≤ 1e-5) as queries to search against the following protein databases: NCBI nr, Swiss-Prot, COSMOSS, KEGG and COG database. Because the unigene sequences have no annotated open reading frames, proteins with highest ranks in BLASTX results were taken to decide the direction and coding region of the assembled transcripts. The best potential coding region of unigenes with no hit in these protein databases were predicted using ESTScan [78] with parameters trained on the annotated unigenes. Subsequently, all predicted coding regions from unigenes were translated into protein sequences using the standard codon table.

According to the NR annotation results, the top BLASTX hits were used to identify putative homologous proteins and annotate each unigene sequence with gene ontology (GO) terms using Blast2GO [48] program. The GO annotation information of *P. patens* genome was downloaded from the *Physcomitrella patens* computational biology resource site (http://www.cosmoss.org). GO classification and comparison with *P. patens* was performed using WEGO [79] according to molecular function, biological process, and cellular component ontologies.

Gene ontology enrichment analysis for the selected gene set with RPKM values [39] above 100 was performed and visualized in Cytoscape (v2.8.3). The cytoscape plugin BiNGO (v2.44) [80] was customized with the *S. caninervis* transcriptome GO annotation results and was then used to perform the hypergeometric statistical test of significance (corrected p-value < 0.05) to assess GO term enrichment. All GO-slim terms found within the gene list more often than expected by chance were highlighted in BiNGO. To adjust for multiple hypotheses testing, a Benjamini & Hochberg False Discovery Rate (FDR) correction was performed.

For Pfam domain/family annotation, the predicted protein sequences were submitted to search against HMM profiles contained in the Pfam database (version 27.0) [50] using HMMER v3.0 [81,82]. To resolve complex overlapping protein domains, only the most significant (lowest E-value) match within the clan was reported. The perl script PfamScan.pl downloaded from the Pfam database ftp server was used for the annotation and Linux tools was applied to parse the annotation results.

The *S. caninervis* transcription factors were predicted using PlantTFDB v3.0 [57]. The putative transcription factors in *S. caninervis* were initially identified, including proteins that contain a DNA binding domain (inferred from Pfam annotation) or gave a positive Blastp hit (E-value ≤ 1e-5) with recorded *P. patens* or *A. thaliana* transcription factors. Deduced polypeptide sequences were then submitted to the PlantTFDB prediction server (planttfdb.cbi.pku.edu.cn/prediction.php) for further classification and validation.

To investigate the metabolic pathway annotation of unigenes, unigenes were aligned using the KEGG database [83], enzyme codes were acquired for each sequence and EC accession numbers were used to color and retrieve the corresponding KEGG pathway maps. Scrutiny of transcript diversity and abundance was performed with MapMan [84,85]. The deduced polypeptide sequences were submitted to Mercator webserver [86] to classify them into MapMan functional plant categories. For color-coded representation (heat map) in MapMan, the log2 transformed of the RPKM-normalized expression counts was used. Deduced polypeptide sequences shorter than 100 amino acids or belonging to the least 5% abundant expressed transcripts (RPKM < 0.17) were not used to generate the MapMan metabolic pathway maps.

For comparison of gene models with *A. thaliana* and *P. patens*, protein-coding gene models in TAIR10 (http://www.arabidopsis.org) and COSMOSS v1.6 (http://www.cosmoss.org) were used. Prior to analysis of PlantOG (Plant Ortholog Groups) memberships, proteins inferred from the *S. caninervis* transcriptome were subjected to CD-HIT [87] (identity ≥0.95 and coverage ≥0.9) to eliminate redundancy. All protein sequences shorter than 50 amino acids were discarded. Protein sequences were clustered using OrthoMCL [65].

## Abbreviations

BSC: Biological soil crust; GO: Gene ontology; RPKM: Reads per kilobase per million mapped reads; TF: Transcription factor.

## Competing interests

The authors declare that they have no competing interests.

## Authors’ contributions

BG performed the bioinformatics analyses and drafted the manuscript. DZ conceived the study and provided financial support for the project. XL contributed to the data analysis. HY participated in plant preparations. AW contributed to data interpretation and revised the manuscript. All authors read and approved the final manuscript.

## Supplementary Material

Additional file 1: Table S1 Comprehensive annotation of *S. caninervis* unigenes. Including BLASTX search hits in NCBI-Nr, Swiss-Prot, COG, KEGG and COSMOSS databases and all annotated transcription factors using PlantTFDB. Click here for file

Additional file 2: Table S2 GO comparison results of *P. patens* genome and *S. caninervis* transcriptome. Click here for file

Additional file 3: Figure S1 Enrichment network based on GO of the most abundantly expressed transcripts (RPKM > 100) in the transcriptome. Significantly overrepresented (p-value < 0.05) GO terms based on GO-slim were visualized in Cytoscape. The node size is in proportional to the number of unigenes in the GO category. The color represents the enrichment significance. Nodes with white color are not enriched but show the essential hierarchical relationship among the enriched GO-slim terms. Click here for file

Additional file 4: Table S3 Pfam domain/family annotation and statistics of *S. caninervis* sequences (2 sheets). Click here for file

Additional file 5: Table S4 Statistics of KEGG pathways annotated in *S. caninervis* transcriptome. Click here for file

Additional file 6 **MapMan overview of *S. caninervis* cellular metabolism.** Individual assembled transcripts are represented by colored squares. The color code scale is based on the log2 of the RPKM values of each unigene. The greater intensity of red is associated with higher transcript abundance. Green highlighted metabolic pathways are biosynthetic while pink highlighted metabolic pathways are degradative. Click here for file
