# Supplementary material for: De novo assembly and characterization of the transcriptome in the desiccation-tolerant moss Syntrichia caninervis
Source: BMC Res Notes. 2014 Aug 3;7:490. doi: 10.1186/1756-0500-7-490 (PMC4124477; doi:10.1186/1756-0500-7-490)
Supplement: Additional file 2: Table S2 — GO comparison results of P. patens genome and S. caninervis transcriptome. [file 1756-0500-7-490-S2.pdf]

Table: GO comparison of *P. patens* genome and *S. caninervis* transcriptome

Ontology	GO-IDs	Description	Number of Gene(Ppa:Sca)	Percentage of Genes(Ppa:Sca)	P-value*
Cellular component	GO:0005576	extracellular region	544:193	2.8:0.8	0
	GO:0005623	cell	15236:16039	79.0:66.3	0
	GO:0031974	membrane-enclosed lumen	840:625	4.4:2.6	0
	GO:0031975	envelope	435:887	2.3:3.7	0
	GO:0032991	macromolecular complex	1887:2904	9.8:12.0	0
	GO:0043226	organelle	9404:10852	48.8:44.9	0
	GO:0044422	organelle part	2228:2988	11.6:12.4	0.011
	GO:0044464	cell part	15235:16039	79.0:66.3	0
Biological process	GO:0000003	reproduction	273:787	1.4:3.3	0
	GO:0008152	metabolic process	8011:11794	41.6:48.8	0
	GO:0009987	cellular process	7971:9617	41.3:39.8	0.001
	GO:0010926	anatomical structure formation	286:280	1.5:1.2	0.003
	GO:0016043	cellular component organization	734:1212	3.8:5.0	0
	GO:0022414	reproductive process	265:781	1.4:3.2	0
	GO:0032501	multicellular organismal process	658:1151	3.4:4.8	0
	GO:0032502	developmental process	600:1598	3.1:6.6	0
	GO:0043473	pigmentation	2135:1366	11.1:5.6	0
	GO:0044085	cellular component biogenesis	446:521	2.3:2.2	0.264
	GO:0050896	response to stimulus	1315:2775	6.8:11.5	0
	GO:0051179	localization	1663:2583	8.6:10.7	0
	GO:0051234	establishment of localization	1631:2508	8.5:10.4	0
	GO:0051704	multi-organism process	200:380	1.0:1.6	0
	GO:0065007	biological regulation	2335:1808	12.1:7.5	0
Molecular function	GO:0003824	catalytic activity	7691:12854	39.9:53.2	0
	GO:0005198	structural molecule activity	716:1158	3.7:4.8	0
	GO:0005215	transporter activity	1013:1488	5.3:6.2	0
	GO:0005488	binding	9460:12874	49.1:53.2	0
	GO:0009055	electron carrier activity	487:06:00	2.5:0.0	0
	GO:0030528	transcription regulator activity	1220:171	6.3:0.7	0
	GO:0045182	translation regulator activity	158:327	0.8:1.4	0
	GO:0060089	molecular transducer activity	339:556	1.8:2.3	0

*WEGO employed the pearson Chi-Square test method to calculate the p-value of the two GO annotation dataset.
