# Supplementary figures and images for: De novo assembly and characterization of the transcriptome in the desiccation-tolerant moss Syntrichia caninervis
Source: BMC Res Notes. 2014 Aug 3;7:490. doi: 10.1186/1756-0500-7-490 (PMC4124477; doi:10.1186/1756-0500-7-490)

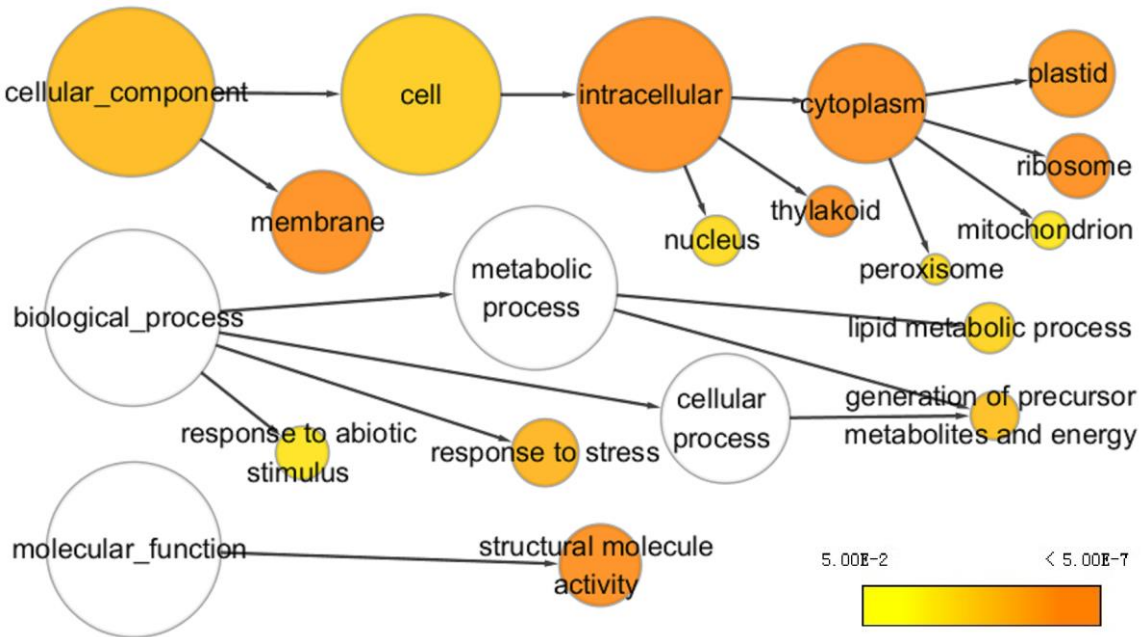

Supplement: Additional file 3: Figure S1 — Enrichment network based on GO of the most abundantly expressed transcripts (RPKM > 100) in the transcriptome. Significantly overrepresented (p-value < 0.05) GO terms based on GO-slim were visualized in Cytoscape. The node size is in proportional to the number of unigenes in the GO category. The color represents the enrichment significance. Nodes with white color are not enriched but show the essential hierarchical relationship among the enriched GO-slim terms. [file 1756-0500-7-490-S3.pdf]

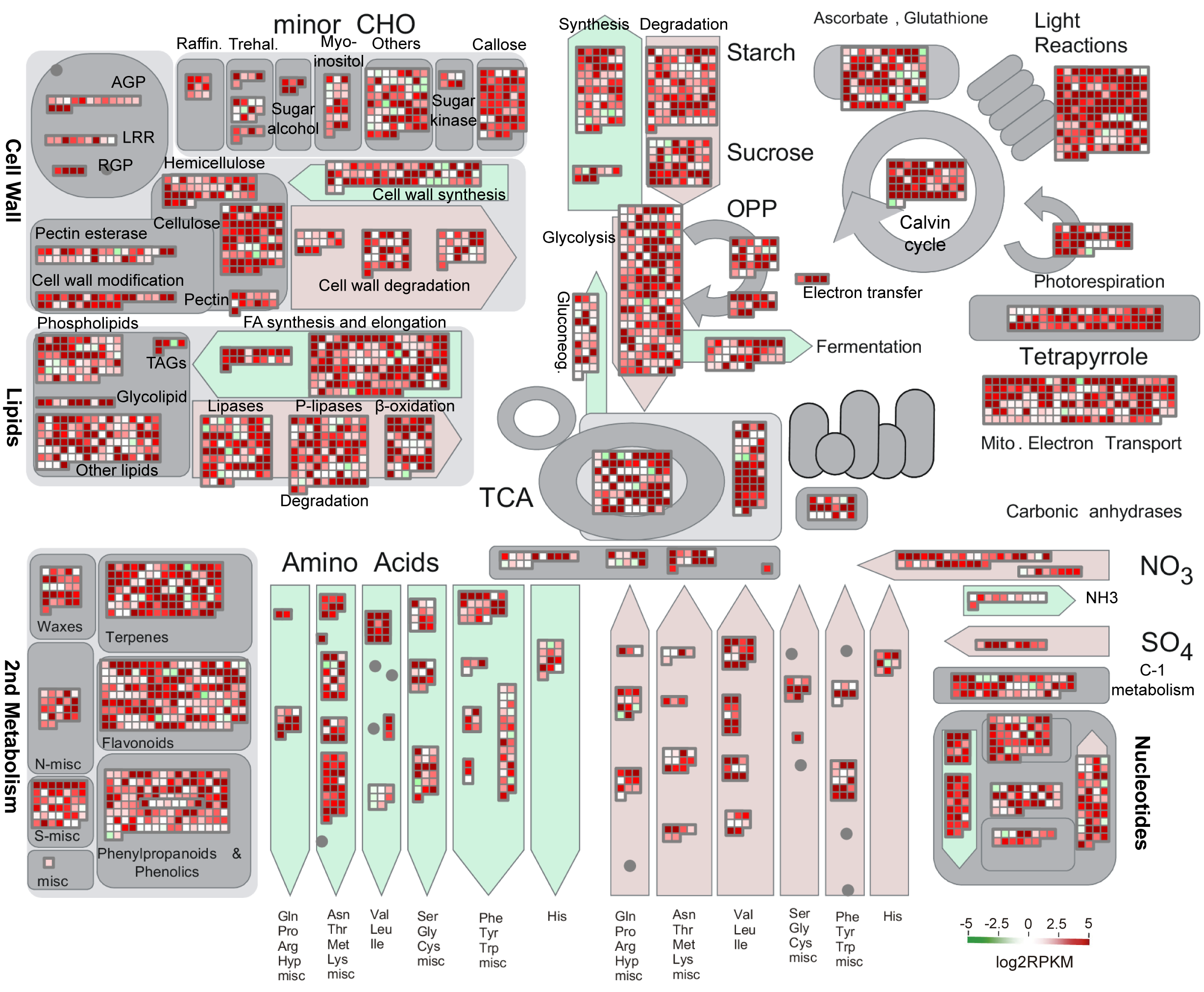

Supplement: Additional file 6 — MapMan overview of S. caninervis cellular metabolism. Individual assembled transcripts are represented by colored squares. The color code scale is based on the log2 of the RPKM values of each unigene. The greater intensity of red is associated with higher transcript abundance. Green highlighted metabolic pathways are biosynthetic while pink highlighted metabolic pathways are degradative. [file 1756-0500-7-490-S6.tiff]
